# Plasma Soluble Urokinase-Type Plasminogen Activator Receptor Is Not Associated with Neurological Outcome in Patients with Aneurysmal Subarachnoid Hemorrhage

**DOI:** 10.3389/fneur.2017.00144

**Published:** 2017-04-18

**Authors:** Heikki Kiiski, Ville Jalkanen, Marika Ala-Peijari, Mari Hämäläinen, Eeva Moilanen, Jukka Peltola, Jyrki Tenhunen

**Affiliations:** ^1^Critical Care Medicine Research Group, Department of Intensive Care, Tampere University Hospital, Tampere, Finland; ^2^The Immunopharmacology Research Group, Faculty of Medicine and Life Sciences, University of Tampere, Tampere University Hospital, Tampere, Finland; ^3^Department of Neurology, University of Tampere, Tampere University Hospital, Tampere, Finland; ^4^Department of Surgical Sciences, Division of Anesthesiology and Intensive Care, Uppsala University, Uppsala, Sweden

**Keywords:** aneurysmal subarachnoid hemorrhage, biomarkers, neurological outcome, secondary brain injury, soluble urokinase-type plasminogen activator receptor, neuroinflammation

## Abstract

**Object:**

Aneurysmal subarachnoid hemorrhage (aSAH) is a common cause of death or long-term disability. Despite advances in neurocritical care, there is still only a very limited ability to monitor the development of secondary brain injury or to predict neurological outcome after aSAH. Soluble urokinase-type plasminogen activator receptor (suPAR) has shown potential as a prognostic and as an inflammatory biomarker in a wide range of critical illnesses since it displays an association with overall immune system activation. This is the first time that suPAR has been evaluated as a prognostic biomarker in aSAH.

**Methods:**

In this prospective population-based study, plasma suPAR levels were measured in aSAH patients (*n* = 47) for up to 5 days. suPAR was measured at 0, 12, and 24 h after patient admission to the intensive care unit (ICU) and daily thereafter until he/she was transferred from the ICU. The patients’ neurological outcome was evaluated with the modified Rankin Scale (mRS) at 6 months after aSAH.

**Results:**

suPAR levels (*n* = 47) during the first 24 h after aSAH were comparable in groups with a favorable (mRS 0–2) or an unfavorable (mRS 3–6) outcome. suPAR levels during the first 24 h were not associated with the findings in the primary brain CT, with acute hydrocephalus, or with antimicrobial medication use during 5-days’ follow-up. suPAR levels were associated with generally accepted inflammatory biomarkers (C-reactive protein, leukocyte count).

**Conclusion:**

Plasma suPAR level was not associated with either neurological outcome or selected clinical conditions. While suPAR is a promising biomarker for prognostication in several conditions requiring intensive care, it did not reveal any value as a prognostic biomarker after aSAH.

## Introduction

Urokinase plasminogen activator receptor (uPAR) (CD87) is present on various immunologically active cells, and its expression becomes elevated by inflammatory conditions and ischemic diseases (1, 2). The soluble form [soluble urokinase-type plasminogen activator receptor (suPAR)] in serum or plasma has emerged as an inflammatory biomarker capable of reflecting overall immune system activation (3, 4).

Previously, it has been shown that uPAR expression is induced in cerebral ischemia (5) and traumatic brain injury (6). Moreover, it has been claimed that uPAR may further augment cerebral injury (7). The induction of uPAR expression on the cell surface is believed to increase the levels of the soluble form of uPAR (8). Previously, suPAR has been shown to have predictive value in acutely, critically ill patients (9–13), including those who have suffered brain trauma (14). However, as far as we are aware, suPAR concentrations have not been evaluated as a prognostic biomarker in patients with subarachnoid hemorrhage.

Aneurysmal subarachnoid hemorrhage (aSAH) is a devastating disease causing long-term disability and up to 50% mortality (15). A significant proportion of the patients are young and previously healthy in comparison to individuals suffering other types of strokes (16). The main causes for poor prognosis are early brain injury and delayed cerebral ischemia (DCI), which cause permanent neurological deficits (17–19). The prediction of outcome is difficult and unsatisfactory as the secondary injury process in aSAH is multifactorial and incompletely understood (17, 20).

It is well established that inflammation plays a major role in vasospasm and subsequent DCI after aSAH (21, 22). A plethora of biomarkers have been studied in aSAH and DCI, e.g., neuron and astrocyte-specific markers (e.g., NSE, s100b, and UCHL-1), inflammatory biomarkers (e.g., IL-6, HMGB-1), and molecular adhesion and extracellular matrix markers (e.g., MMP-9) (16, 23–25). However, none of these putative biomarkers has so far proved to be useful in clinical decision-making. As ischemic events and inflammation are one characteristic feature of aSAH, it seemed reasonable to speculate that circulating plasma suPAR concentrations would increase during the acute stage after aSAH. Therefore, we hypothesized that either the plasma suPAR concentration or alternatively its changes over time could be useful in predicting the neurological outcome following aSAH.

## Materials and Methods

The clinical data and blood samples from this patient cohort have been used in a previously published study (25). Following registration in Clinical Trials (NCT02026596, https://clinicaltrials.gov) and approval by the institutional ethics committee, we conducted a prospective, observational, single-center clinical study in Tampere University Hospital (Tampere, Finland) intensive care unit (ICU). The study population consisted of 61 consecutive adult aSAH patients admitted to our tertiary referral center during a 10-month period in 2013. Written informed consent was obtained from each of the patients or from their next of kin. All patients were treated according to standard in-house guidelines, which included intravenous nimodipine to prevent vasospasm and routine laboratory samples. In the final analyses, we chose to exclude those 14 patients with an unknown time of onset of symptoms or in whom the suPAR concentration was not measured during the first 24 h after the onset of symptoms. By including only those 47 patients with a known onset of clinical ictus and suPAR measurement during the first 24 h after the onset of symptoms, we eliminated the possibility that changes in suPAR concentrations would be attributable to different delays to hospital admission after aSAH.

The plasma suPAR concentration was measured at 0, 12, and 24 h after the admission and every 24 h for up to 5 days or until the patient was transferred from the ICU. World Federation of Neurological Surgeons Grading Scale, Fisher grade, and 6-month modified Rankin Scale (mRS) were used to evaluate the severity of aSAH and neurological recovery as previously described (25). The incidence of acute hydrocephalus was defined as the need for ventriculostomy on a clinical basis during the first 24 h after aSAH. Infection was defined as the need for antimicrobial medication during intensive care follow-up period.

As a part of our in-house guideline, an arterial cannula was routinely inserted. Blood samples for suPAR were collected into EDTA-containing tubes from the arterial cannula and the samples were immediately centrifuged for 10 min at 2,000 *g* at room temperature. After centrifugation, the plasma was collected and frozen at −70°C. After thawing, plasma suPAR levels were measured with a commercially available enzyme-linked immunosorbent assay kit according to the manufacturer’s instructions (suPARnostic^®^, ViroGates, Birkeroed, Denmark). The detection limit and inter-assay coefficient of variation were 0.45 ng/ml and 3.2%, respectively.

Before the statistical analysis, the suPAR measurements were divided into consecutive 24 h intervals, starting from the onset of symptoms. If suPAR was measured more than once per interval, the mean concentration was used. In a subgroup of 22 patients who had up to 5-days’ follow-up, we also checked whether the patient had been treated for DCI. Initiation of this treatment was based on clinical evaluation. Statistical analyses were performed with R (version 3.3.2 for Mac Os X). Fisher’s exact test was used with the categorical variables. Due to the non-normal distribution of measured biomarkers, Mann–Whitney *U*-test was used for between-group comparisons. Correlations were evaluated with Spearman’s correlation test. Linear regression was used in the time interval analyses.

## Results

**Figure 1:**
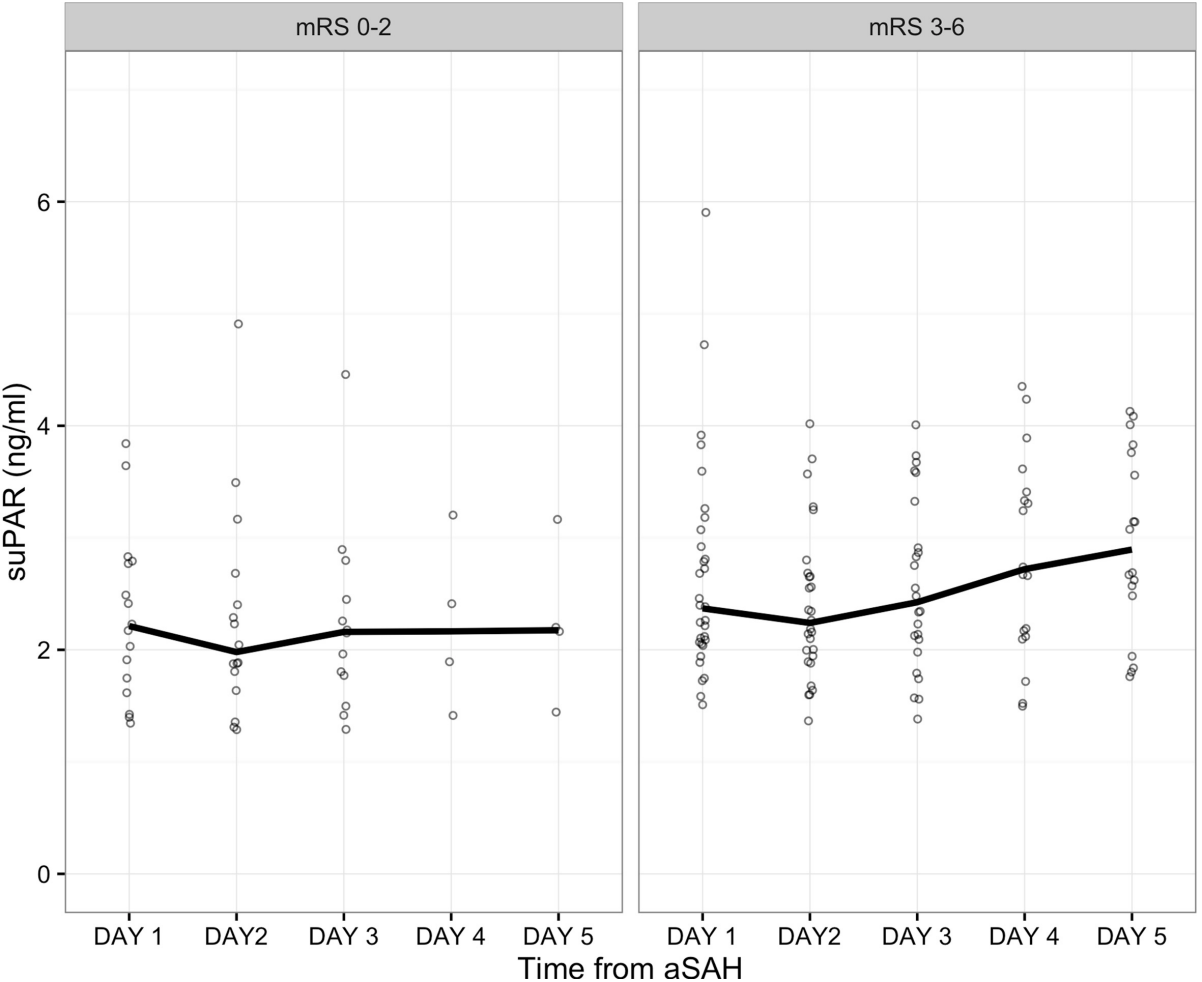
**Soluble urokinase-type plasminogen activator receptor (suPAR) levels in all patients and at all time intervals**. Values are grouped according to whether the patients had a favorable or an unfavorable neurological outcome. Dots represent individual patient values. The line represents group median.

The basic characteristics of the study cohort and the subset of patients with 5-day follow-up have been previously reported (25). The time course of plasma suPAR concentrations in the two groups according to neurological outcome are depicted in Figure 1.

**Figure 2:**
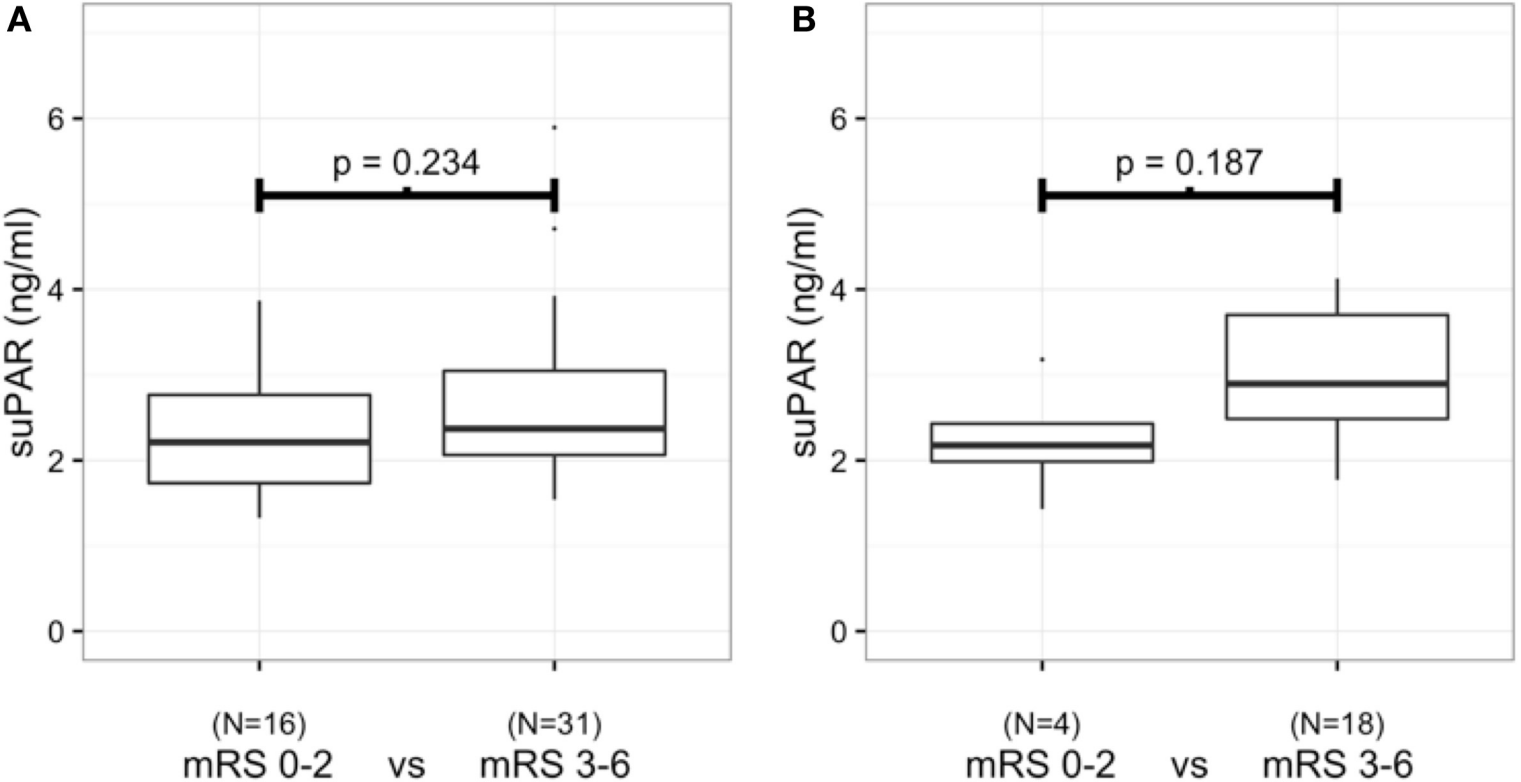
**Soluble urokinase-type plasminogen activator receptor (suPAR) levels during the first 24 h after aneurysmal subarachnoid hemorrhage between patients (*n* = 47) with a favorable or an unfavorable neurological outcome (A)**. suPAR levels at the end of 5-day follow-up between patients (*n* = 22) with a favorable or an unfavorable neurological outcome **(B)**.

Plasma suPAR concentrations during the first 24 h after aSAH were comparable in the groups with a favorable (mRS 0–2) and an unfavorable (mRS 3–6) outcome (Figure 2A). Similarly, no differences were detected in the suPAR levels between those patients presenting with severe (WFNS 4–5) vs. non-severe (WFNS 1–3) findings in terms of their clinical status on admission (Table 1). Furthermore, the plasma suPAR concentration during the first 24 h was not associated with the findings from the primary brain CT (Fisher grade) with acute hydrocephalus or with antimicrobial medication during the 5-days’ follow-up (Table 1). Age over 70 years was a strong predictor of an unfavorable neurological outcome, i.e., only one patient over 70 years experienced a neurologically favorable outcome (*p* = 0.037, Table 2).

**Table 1:** **Soluble urokinase-type plasminogen activator receptor (suPAR) concentrations (*n* = 47) within 24 h from aneurysmal subarachnoid hemorrhage and the association with selected clinical conditions**.

suPAR (ng/ml)	Mean	SD	Median	IQR	*p*-Value
Modified Rankin Scale					0.234
0–2 (*n* = 16)	2.30	0.75	2.21	1.73–2.77	
3–6 (*n* = 31)	2.66	0.96	2.37	2.06–3.05	
World Federation of Neurological Surgeons grading scale					0.803
1–3 (*n* = 28)	2.59	1.05	2.26	2.00–2.79	
4–5 (*n* = 19)	2.47	0.66	2.41	2.00–2.86	
Fisher					0.240
1–2 (*n* = 14)	2.36	0.93	2.05	1.67–2.78	
3–4 (*n* = 33)	2.61	0.90	2.41	2.07–2.80	
Infection					0.402
Yes (*n* = 14)	2.55	0.64	2.61	2.21–2.88	
No (*n* = 33)	2.53	1.01	2.22	1.92–2.79	
Acute hydrocephalus					0.845
Yes (*n* = 19)	2.54	0.83	2.27	2.09–2.80	
No (*n* = 28)	2.53	0.97	2.34	1.92–2.83	

*Mann–Whitney U-test was used*.

**Table 2:** **The relationship between neurological outcome and age**.

	Age ≤ 70	Age > 70	*p*-Value
			0.037
Modified Rankin Scale (mRS) 0–2	15	1	
mRS 3–6	20	11	

*Fisher’s exact test was used*.

**Figure 3:**
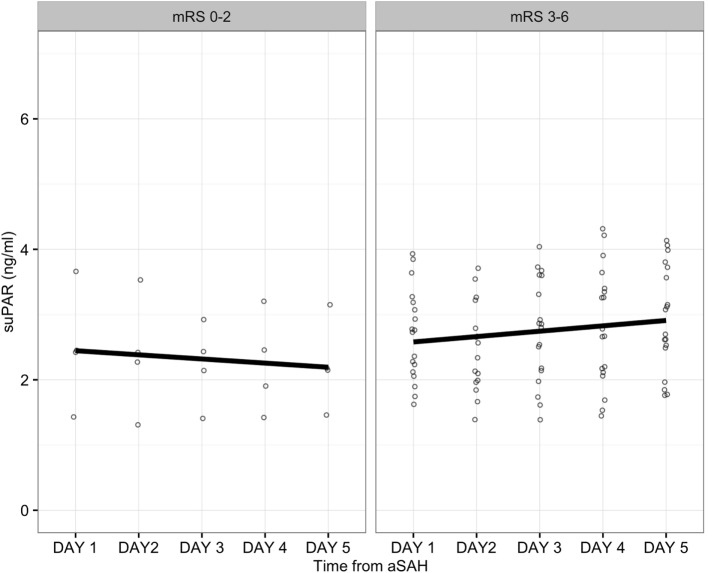
**Soluble urokinase-type plasminogen activator receptor (suPAR) levels for patients with a follow-up of up to 5 days (*n* = 22)**. Dots represent individual patient values. Regression line is calculated with linear regression. Values are grouped according to favorable (*p* = 0.584) or non-favorable (*p* = 0.158) neurological outcome.

suPAR was measured daily up to 5 days after the admission unless the patient died or was transferred from the ICU. In the 22 patients in whom we had suPAR concentrations measured up to 5 days, four patients achieved a favorable neurological outcome (mRS 0–2). The linear regression did not detect any statistically significant elevation of suPAR levels during the 5-days’ follow-up in patients with either a favorable (mRS 0–2) or an unfavorable (mRS 3–6) neurological outcome (Figure 3). In addition, at day five, plasma suPAR concentrations were comparable in the two groups (Figure 2B).

Delayed cerebral ischemia treatment was initiated in 16/22 patients during the hospital stay in the subgroup in which we had at least a 5-day ICU follow-up. Neither DCI treatment nor infection or acute hydrocephalus was associated with plasma suPAR concentrations (Table 3).

**Table 3:** **Soluble urokinase-type plasminogen activator receptor (suPAR) levels on day five from aneurysmal subarachnoid hemorrhage patients (*n* = 22) in whom there was 5-days’ intensive care unit follow-up data and their association with selected clinical conditions**.

suPAR (ng/ml)	Mean	SD	Median	IQR	*p*-Value
Modified Rankin Scale					0.187
0–2 (*n* = 4)	2.24	0.72	2.18	1.99–2.43	
3–6 (*n* = 18)	2.95	0.81	2.90	2.49–3.70	
Delayed cerebral ischemia treatment					0.854
Yes (*n* = 16)	2.80	0.79	2.64	2.18–3.32	
No (*n* = 6)	2.89	1.00	3.12	2.25–3.48	
Infection					0.511
Yes (*n* = 11)	2.97	0.81	3.09	2.60–3.67	
No (*n* = 11)	2.68	0.86	2.46	2.01–3.17	
Acute hydrocephalus					0.511
Yes (*n* = 11)	2.70	0.91	2.64	1.91–3.45	
No (*n* = 11)	2.95	0.77	3.09	2.32–3.39	

*Mann–Whitney U-test was used*.

The peak plasma suPAR concentration during the 5 days of follow-up was positively correlated with peak levels of C-reactive protein (CRP) (*p* = 0.039) and leukocyte numbers (*p* = 0.006). The plasma suPAR concentration was positively correlated with the leukocyte count during the first 24 h (*p* = 0.049). In contrast, suPAR levels were not associated with age (Table 4).

**Table 4:** **Soluble urokinase-type plasminogen activator receptor and its association with leukocyte count, C-reactive protein (CRP), and age**.

	Spearman rho	*p*-Value
**Day 1 (*n* = 47)**		
Leukocyte count	0.289	0.049
CRP	0.261	0.077
AGE	0.228	0.123
**5-day intensive care unit follow-up (*n* = 22)**		
Maximum leukocyte count during follow-up	0.568	0.006
Maximum CRP during follow-up	0.443	0.039
Age	0.143	0.527

## Discussion

The present study aimed to evaluate the potential prognostic value of plasma suPAR concentrations after an aSAH. In contrast to our working hypothesis, plasma suPAR did not show any association with neurological outcome, survival, acute hydrocephalus, clinical infection, or DCI in our patient cohort.

The inflammatory reaction and increase of systemic inflammatory mediators in aSAH is well documented (22, 26). Even though substantial evidence is accumulating in the literature highlighting the significant role of neuroinflammation in the outcome of aSAH, it is still unclear which, if any, inflammatory biomarkers can be used to guide clinical decision-making. The apparent biphasic nature of the inflammatory response after aSAH makes this challenge even more demanding. Neuroinflammation seems to have properties, which can be considered in some cases as protective, but in others, as deleterious, e.g., depending on the magnitude of the response, time-point after the ictus when activation of the inflammatory response occurs, and the type of cells recruited in the response (27). Hence, it is not surprising that there is inconsistency regarding the prognostic value of many inflammatory biomarkers such as IL-6 and HMGB1 after aSAH (19, 25, 28, 29). Nevertheless, although no biomarker has been identified, these studies have increased our understanding of the inflammatory process in aSAH and, in fact, also novel inflammatory biomarkers are claimed to have some prognostic potential, e.g., toll-like receptor 4 (30).

suPAR is considered as an inflammatory biomarker and mediator, a proposal that is well supported in the literature. Previously, increased serum or plasma suPAR levels have been postulated as a prognostic factor for poor outcome in critically ill patients with an inflammatory condition (31). The plasma suPAR concentrations in our aSAH cohort were low compared to septic and non-septic ICU patients with organ dysfunction (32). Nosocomial infections, organ dysfunction, and SIRS are frequent after aSAH (33, 34). Although the value of suPAR has been verified in infections and organ dysfunction (13, 32, 35), we did not detect high levels of suPAR, even later in the course of intensive care. One possible confounding factor distinguishing aSAH from other acute neurological conditions is that all of our patients received nimodipine to prevent DCI. Nimodipine has been shown to decrease plasminogen activator inhibitor 1 (PAI-1) activity (36). As PAI-1 is the major inhibitor of urokinase plasminogen activator (uPA), plasminogen activity and fibrinolysis may increase as a consequence of decreased PAI-1 activity. UPA can cleave the GPI-anchor on cell surface, but since a correct ratio of uPA is required for cleavage, it is possible that excess uPA due to nimodipine may inhibit the cleavage of suPAR from the cell surface (4, 8). suPAR also displays uPA dose dependence for binding to vitronectin (37), which may alter suPAR levels. Furthermore, previous reports have described suPAR-fragment release from activated neutrophils (38) and inhibition of neutrophil activation by two calcium antagonists, felodipine, and nimodipine (39), thereby supporting the concept that nimodipine may indeed be the factor modifying the suPAR response in our patient cohort. This speculative hypothesis and thus the potential direct effect of nimodipine on plasma suPAR concentrations could not be further tested/evaluated in our patient cohort. Moreover, as nimodipine is considered as part of current best practice, it would be unethical to establish a control group not receiving nimodipine after aSAH. Any further experiments to test this hypothesis will need to be conducted as preclinical/animal studies.

Overall, the suPAR response, i.e., the increase in the plasma concentrations of suPAR observed in this study, was rather modest in comparison with that observed in other critically ill patients, even though there was biochemically logical correlation between suPAR levels and generally accepted inflammatory biomarkers (CRP, leukocyte count). The correlation, however, was weak in comparison to previously reported results (35, 40) possibly indicating that there are numerous factors influencing inflammatory biomarkers and mediators in aSAH. In addition, although higher age was associated with poor outcome *per se*, in our patient cohort, we observed no correlation between age and plasma suPAR levels. This finding contradicts the results of several previous studies (10, 13, 41, 42). Finally, the low incidence (29.8%) of nosocomial infections in our patient cohort may partially explain the observed low plasma suPAR levels.

Even though serum suPAR levels have been shown to be elevated in ischemic stroke (43) and in cerebrospinal fluid (CSF) following disruption of the blood–brain barrier (44), no marked elevation of plasma suPAR was found in patients either diagnosed with DCI or acute hydrocephalus. Further analyses will be necessary to clarify potential importance of suPAR release to CSF in patients with DCI and acute hydrocephalus. In order to reveal the actual role of suPAR as a biomarker or mediator in aSAH, it would be worthwhile evaluating the potential value of suPAR levels in CSF in diagnosing ventriculitis related to ventriculostomy catheter.

Our study has some limitations. First, we had some patients that were lost to follow-up. In other words, we were not able to obtain samples late in the course of the acute illness as patients had either died or been transferred to some other health-care facility. These patients represent two extremes, i.e., either the best or the worst outcome, and this may have altered the results of our analysis. Second, our sample size was limited. In particular, only four patients with a favorable neurological outcome remained in the final analyses on day 5. However, some of our patients experienced mild whereas others had very severe presentations of aSAH. We followed suPAR levels during the whole ICU stay and suPAR levels were constantly low with no high peaks being observed. This suggests that aSAH does not induce high suPAR levels in plasma or they are depressed by some aspect of the treatment, for example, administration of the calcium antagonist. Third, our study is a single-center study. Although our unit is a tertiary referral hospital with a high patient influx, single-center bias is possible. Despite their relatively low numbers, it is of interest that those patients with a good neurological outcome had remarkably low plasma suPAR levels with a very small SD (Figure 2B). Our previous studies have suggested that while high suPAR levels may be prognostic for poor outcome, in contrast, a low plasma suPAR concentration is predictive of a good outcome (11, 45). In the present study, the number of patients is limited, but the same phenomenon may apply to aSAH.

## Conclusion

This study reports the first population-based prospective, observational results evaluating plasma suPAR concentrations in aSAH. Plasma suPAR levels were not associated with neurological outcome or selected clinical conditions. While suPAR is a promising biomarker in several conditions requiring intensive care, based on this study, it does not seem to be useful as a prognostic biomarker in patients with aSAH.

## Ethics Statement

The study was approved by the Ethics Committee of Pirkanmaa Hospital District. Written informed consent was obtained from each of the patients or from the next of kin.

## Author Contributions

HK contributed to study conception, neurological outcome evaluations, statistical analyses, and drafted of the manuscript. VJ contributed to patient recruitment, statistical analyses, and drafted the manuscript. MA-P contributed to patient recruitment, evaluation of the initial clinical severity, evaluation of the initial computed tomography findings, and drafted the manuscript. MH contributed to the laboratory analysis of suPAR and drafted the manuscript. EM contributed to the laboratory analysis of suPAR and drafted the manuscript. JP contributed to neurological outcome evaluations, and drafted the manuscript. JT contributed to study conception, statistical analyses, and drafted the manuscript.

## Conflict of Interest Statement

The authors declare that the research was conducted in the absence of any commercial or financial relationships that could be construed as a potential conflict of interest.
